# Nutrition and Obesity in the Pathogenesis of Youth-Onset Type 1 Diabetes and Its Complications

**DOI:** 10.3389/fendo.2021.622901

**Published:** 2021-03-22

**Authors:** Christine A. March, Dorothy J. Becker, Ingrid M. Libman

**Affiliations:** Division of Pediatric Endocrinology and Diabetes, University of Pittsburgh, UPMC Children’s Hospital of Pittsburgh, Pittsburgh, PA, United States

**Keywords:** type 1 diabetes, obesity, nutrition, double diabetes, cardiovascular complications

## Abstract

Since the 1980s, there has been a dramatic rise in the prevalence of overweight and obesity in pediatric populations, in large part driven by sedentary lifestyles and changing dietary patterns with more processed foods. In parallel with the rise in pediatric obesity in the general population, the prevalence of overweight and obesity has increased among children and adolescents with type 1 diabetes. Adiposity has been implicated in a variety of mechanisms both potentiating the risk for type 1 diabetes as well as exacerbating long-term complications, particularly cardiovascular disease. Treatment options targeting the unique needs of obese pediatric patients, both before and after diagnosis of type 1 diabetes, are limited. In this review, we discuss the history of the epidemiology of the obesity epidemic in the context of pediatric type 1 diabetes, highlight the possible role of obesity in type 1 diabetes pathogenesis and review the concept of “double diabetes”. The impact of obesity at and after diagnosis will be discussed, including noted differences in clinical and biochemical markers, lipid abnormalities, and long-term cardiovascular complications. Finally, we will review the existing literature on pharmacologic and nutritional interventions as potential treatment strategies for youth with coexisting type 1 diabetes and obesity.

## Introduction 

Autoimmune type 1 diabetes is one of the most common chronic conditions of childhood, with the incidence increasing worldwide (1–3). Our understanding of the diverse forms of diabetes has evolved since the 1950s, with several different classification systems proposed until the most recent distinction of type 1 and type 2 diabetes in the 1990s (4). This classification focused on pathogenesis, separating diabetes resulting from absolute insulin deficiency, typically secondary to autoimmunity, or from progressive loss of insulin secretion in the setting of insulin resistance. However, continued advances have unraveled the variable genetic, immunologic, and metabolic factors that contribute to diabetes, and our current concept of type 1 diabetes in youth acknowledges significant heterogeneity of this disease. One noteworthy factor affecting this disease is the rise in childhood overweight and obesity (5). A known risk factor for insulin resistance, obesity is now a frequently recognized comorbidity in type 1 diabetes and may compound both the risk for and subsequent complications of type 1 diabetes in youth.

In this review, we highlight the epidemiological trends in obesity and new onset type 1 diabetes, including possible etiological explanations. With the rising prevalence of overweight and obesity at diagnosis of type 1 diabetes and persisting over time, we will address three important questions. First, does obesity contribute to the increased incidence of type 1 diabetes in youth and how? Second, what are the short- and long-term impacts of obesity on type 1 diabetes and its complications? And finally, can targeted treatment strategies optimize outcomes in youth with coexisting obesity and type 1 diabetes?

## Changing Trends in Diabetes Epidemiology

### Parallel Epidemics: Obesity and Type 1 Diabetes

The obesity epidemic is one of the defining global public health issues of our time. Over the latter half of the 20^th^ century, the prevalence of overweight and obesity in pediatric populations increased dramatically in the United States. Approximately one-third of American children are overweight or obese (6). Recent estimates among children age 2–19 years point to 17% of children with obesity [body mass index (BMI) ≥ 95^th^ percentile] and 5.8% with extreme obesity (BMI at or above 120% of the sex-specific 95^th^ percentile), with some leveling-off in the prevalence of obesity in younger age groups in recent years (5). Similar trends in pediatric overweight and obesity have been observed worldwide (7, 8), though the rates of obesity in the developing world continue to accelerate (9). At the heart of the obesity epidemic are sedentary lifestyles and the “westernized diet”, consisting of increased processed foods, refined sugars, and saturated fat. Metabolic derangements resulting from this diet promote weight gain and contribute to cardiovascular disease, diabetes, and cancer (10). Ultra-processed foods, both nutrient poor and calorically dense, are increasingly linked to higher risk for all-cause mortality in the United States and other countries (11, 12). Added sugars in processed foods are a significant portion of the diet of American children beginning at a young age (13), a concerning finding given the evidence for long-term health problems. Longitudinal data links obesity in childhood to adverse health outcomes in adulthood, including hypertension, fatty liver disease, dyslipidemia, and type 2 diabetes (14–16). In addition to the significant morbidity and mortality associated with adult obesity, there are enormous economic costs that are projected to continue to increase over the next decades (17).

In parallel with the obesity epidemic, the incidence of type 1 diabetes in youth has also been increasing worldwide (1–3). The multi-center SEARCH for Diabetes in Youth Study reported a 1.8% per year increase in the incidence of type 1 diabetes from 2002–2012 adjusted for age, sex, and race/ethnicity; the incidence was higher for racial/ethnic minorities at 4.2% increase per year in Hispanic youth and 2.2% increase per year in African American youth (18). In Europe, the EURODIAB study has examined trends in incidence from 1989 to 2013. Initial findings demonstrated an incidence of 3.2–4.1% per year from 1989–2003 with a subsequent leveling off; however, a follow-up investigation found a persistent and steady rise in the incidence of type 1 diabetes in more recent years of approximately 3% per year, suggesting possible cyclicity in a changing incidence rate (19, 20). No clear etiologic factor has been identified to explain this pattern. Various environmental triggers have been explored, including pathogens, nutritional changes, and obesity (21), though the increasing incidence of type 1 diabetes has continued despite some slowing in the pediatric obesity epidemic in developed countries (9).

### Obesity in Children and Adolescents With Type 1 Diabetes

With the increasing incidences of both type 1 diabetes and obesity, the prevalence of overweight and obesity in youth with type 1 diabetes at diagnosis has also increased. Unlike type 2 diabetes, where obesity is a known risk factor, children with new-onset type 1 diabetes in the past were not overweight at diagnosis and traditionally thought to be thin. Libman et al. was one of the first to identify the changing presentation of youth with diabetes in a study examining new onset insulin dependent diabetes in cohorts of children from 1979–1998 (22). Over a 20-year period, the prevalence of overweight and obesity at diagnosis increased threefold, from 12.6% in the first decade to 36.8% in the second decade. The prevalence of overweight and obesity at onset increased nearly five times among those with confirmed autoimmunity from the 1980s to the 1990s, suggesting that the observation was not driven by increasing cases of type 2 diabetes. Subsequent studies have similarly identified a rise in BMI at diagnosis of type 1 diabetes over time (23–25).

**Figure 1:**
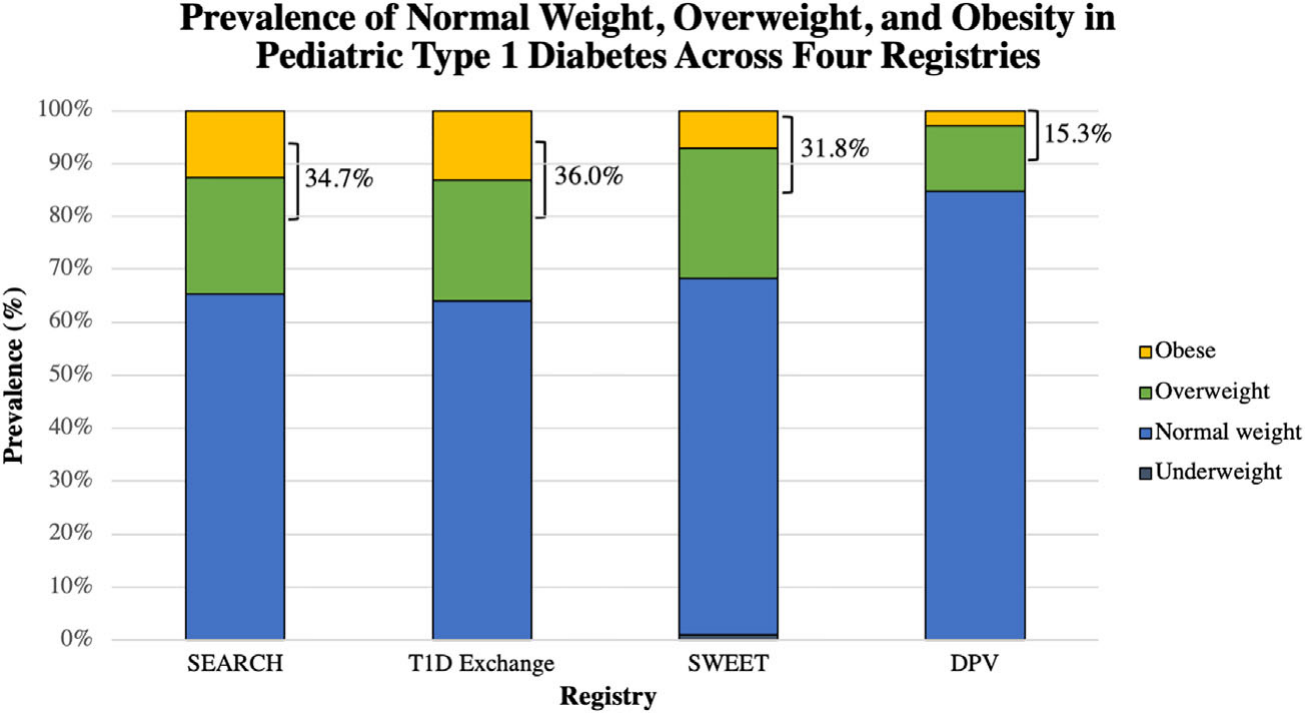
Graphical representation of the proportion of reported normal weight, overweight, and obese youth with type 1 diabetes in each of these four registries: SEARCH (30), T1D Exchange (31), SWEET (32), and the DPV (33). The combined percentage of overweight and obese is shown. The proportion of youth who were underweight was available only for the SWEET registry. SWEET data was reported separately for males and females; a weighted average was obtained and is reported here.

Following diagnosis, several groups have examined the prevalence of overweight and obesity cross-sectionally in children and adolescents with type 1 diabetes at least one year into diagnosis. In single-center reports, the estimated prevalence ranged from 25–40% of youth, with most approaching 35%, in children with a mean duration of diabetes of 5.6–8.7 years (26–29). In recent years, larger, registry-based assessments have confirmed these findings, as illustrated in **Figure 1**. The US-based SEARCH for Diabetes in Youth Study found that 34.7% of participants with type 1 diabetes were overweight or obese (30); another United States-based multicenter collaborative, the Type 1 Diabetes Exchange, estimated this prevalence to be 36% in their population with a mean duration of 6.8 ± 4.1 years (31). In contrast, the European-based, prospective Diabetes Patienten Verlaufsdokumentation (DPV) Registry estimated a lower prevalence of 15.3% of children being overweight/obese in their population with a mean duration of 4.7 ± 3.0 years (33). Most recently, the SWEET registry, an international consortium consisting of 26 countries, found the prevalence to be 29.6% of male patients and 34% of female patients who had a mean duration of diabetes approximately 5.5 years (32). The higher preponderance of overweight/obesity among females has been noted in other studies (26, 29–31, 33, 34), but is not a consistent finding. In the United States, racial/ethnic minorities with type 1 diabetes are more likely to be overweight/obese (30, 31) consistent with national trends in BMI by race/ethnicity among youth without diabetes (35). Importantly, the prevalence of obesity appears to be similar to youth without diabetes in most cases (23, 26, 29), though some have suggested that it may be higher among youth with type 1 diabetes (27, 34).

Several plausible explanations have been reported to explain the rise in obesity in youth with type 1 diabetes. Certainly, this trend mirrors the worldwide epidemic of obesity, and youth with diabetes are likely not immune to the westernized diet despite frequent nutrition counseling. Indeed, children and adolescents with type 1 diabetes generally do not meet the American Diabetes Association (ADA) and International Society for Pediatric and Adolescent Diabetes (ISPAD) dietary recommendations. More than a decade ago, our group found that adolescents with type 1 diabetes consume a higher percentage of their total calories from fat compared to peers without diabetes (36). A subsequent systematic review confirmed that children with type 1 diabetes tend to eat diets higher in saturated fat and also lower in fruits, vegetables, and whole grains, than recommended (37). These findings were echoed in more recent studies examining dietary habits in younger children (38) and adolescents (39) with type 1 diabetes. Diets with a higher percentage of total energy intake coming from fat rather than carbohydrates contribute to both weight gain and, ultimately, adverse lipid profiles (40). Carbohydrate counting, a system by which the insulin dose is calculated based upon the planned number of carbohydrates to be consumed, became widely popularized in the 2010s to increase dietary choice and flexibility for individuals with type 1 diabetes. Whether carbohydrate counting itself has contributed to weight gain is met with some controversy, with studies finding conflicting results (41). A recent study by Donzeau et al. examined carbohydrate counting versus fixed dose insulin for meals and found no change in BMI z-score in youth over one year of follow-up, though the fairly low starting mean BMI z-score (0.7 ± 0.8) of this population and possible selection bias may limit the generalizability of the findings (42).

In addition to diet, inadequate physical activity may also impact glycemia and weight in youth with type 1 diabetes. The guidelines recommend 60 min of moderate to vigorous physical activity per day (43); however, a large portion of youth with diabetes do not meet these guidelines (44). Similar to children without diabetes, various home, peer, and environmental factors may affect physical activity in youth with type 1 diabetes (45), though fear of hypoglycemia and the need for adequate supervision to monitor blood glucoses may be additional considerations (46). Regular activity has been shown to lower hemoglobin A1c, with additional benefits on weight, cardiovascular risk, and well-being (46). In the T1D Exchange cohort, youth who rated themselves as active (defined as 5–7 days/week) rather than inactive (defined as 0–1 days/week) were less likely to be overweight (OR 0.72, 95% CI 0.54–0.96) or obese (OR 0.70, 95% CI 0.49–0.99) in univariate analyses. Despite the evidence that children and adolescents with type 1 diabetes may not follow a healthy diet or get sufficient physical activity, we lack sophisticated studies examining temporal trends in relation to weight gain in this population, specifically.

An additional factor is our revised approach to treatment of type 1 diabetes, specifically with insulin therapy. The Diabetes Control and Complications Trial (DCCT) definitively identified intensive insulin therapy as the gold standard of treatment to prevent the development and progression of long-term micro- and macrovascular complications (47–49). As a result, both increased doses and frequency of dosing of insulin have become standard of care. In the 18 years following the DCCT as part of the Epidemiology of Diabetes Interventions and Complications (EDIC) trial, the prevalence of overweight increased by 47% and obesity increased seven times to 22.7% among participants (50). Prior randomization to the intensive insulin therapy group was predictive of elevated BMI. More recent studies have similarly correlated higher insulin dose and continuous subcutaneous insulin infusion with increased weight gain (28, 31, 33); in one circumstance, each unit increase in hemoglobin A1c decreased the odds of overweight by 8% (OR 0.92, 95% CI 0.87 0.97) (31), again suggesting that intensive treatment to improve glycemic control can result in weight gain. There are likely two explanations. With more insulin, the body is better able to utilize the calories from the food consumed, and in the setting of poor dietary choices and sedentary behavior, this can contribute to weight gain. Another potential adverse effect of intensive insulin therapy, hypoglycemia, may also result in excessive carbohydrate consumption to either treat or prevent low blood sugars, further adding to weight gain (51). This has improved with long-acting insulin analogues and insulin pumps, and may improve further with newer hybrid closed loop technologies, which can minimize hypoglycemia through automated basal rate adjustment (52, 53).

## Obesity and the Pathogenesis of Type 1 Diabetes

### The Accelerator Hypothesis and Double Diabetes

With the changing prevalence of obesity at onset of type 1 diabetes in youth, it has become apparent that there are youth presenting with overlapping characteristics of the different types of diabetes, including features of both autoimmunity and insulin resistance, described as “double diabetes”. Data from our group suggested a higher than expected frequency of a family history of type 2 diabetes in youth with type 1 diabetes from our Children’s Hospital of Pittsburgh Diabetes Registry, leading us to postulate that the increased insulin demands of obesity may accelerate the presentation of autoimmune type 1 diabetes (54, 55). In further support of this theory, we later observed features of insulin resistance, including obesity and acanthosis nigricans in islet autoantibody positive youth with new onset insulin-dependent diabetes (56). A similar conclusion was drawn by T. Wilkin who developed the “accelerator hypothesis”, which proposes that obesity-induced insulin resistance places increased burden on islets in genetically at-risk individuals, inducing autoimmune destruction and/or accelerating its course (57). These hypotheses have called into question the traditional classification structure of type 1 and type 2 diabetes, considering them instead to be two extremes on a spectrum of disease. Wilkin argued that the primary factor differentiating diabetes type is the *tempo* of progression to overt clinical disease, driven by the interplay between β-cell reserve and insulin sensitivity. This phenomenon of double diabetes, has also been described as developing after onset of diabetes. A series of case reports have described combined features of autoimmunity and insulin resistance in children, including a notable case from our group describing a 5-year-old child diagnosed with type 1 diabetes who ultimately developed obesity, acanthosis nigricans, and severe insulin resistance (58). Indeed, youth-onset type 1 diabetes is characterized by significant disease heterogeneity which may in part relate to weight. The complex relationship between environmental factors and genetic risk for type 1 or 2 diabetes likely plays a role in autoimmunity pathogenesis and presentation of clinical disease.

### Clinical Studies Implicating Obesity in Autoimmunity and Type 1 Diabetes

Various studies have investigated the role of birth weight, weight gain, and obesity in the development of islet autoimmunity, progression from single to multiple antibodies, and development of type 1 diabetes. The earliest analyses included population-based cohort and epidemiological case-control studies which correlated a diagnosis of type 1 diabetes with elevated birth weight and early childhood weight gain in infancy as compared to a referent population (59–62). However, these findings were not universal across all populations studied, as others found no supportive evidence linking weight and presentation of type 1 diabetes (63, 64). Subsequently, several prospective cohort studies from birth identified a higher rate of early weight gain and absolute BMI z-score as predictors for the development of islet autoimmunity (65–69). Notably, The Environmental Determinants of Diabetes in the Young (TEDDY) study, a multi-country cohort following children at-risk for type 1 diabetes based upon their HLA-DR-DQ genotype, has examined the associations between weight in the first few years of life with progression from single to multiple antibodies and subsequent development of type 1 diabetes. Weight z-scores at 12 and 24 months were associated with increased risk for progression to multiple antibodies (67), and a higher rate of weight gain in early childhood was associated with progression from autoimmunity to type 1 diabetes in those with the initial presenting autoantibody being directed against glutamic acid decarboxylase (69). Additionally, there may be some relationship between weight gain and earlier age at presentation of type 1 diabetes (23, 61). These studies provide some support for the accelerator hypothesis by suggesting that birth weight and excessive weight gain in early years may hasten diabetes onset, though with little evidence for an association with the onset of islet autoimmunity.

Additional investigations have examined the possible connection between BMI with the progression of type 1 diabetes after autoimmunity onset in older children and adults. Initial comparisons of autoantibody status by BMI at diagnosis were inconclusive. A cross-sectional assessment of 263 children under age 19 years at onset found no significant association between the number of antibodies and measures of adiposity, including BMI and waist circumference (70). A second study examining the TrialNet Pathway to Prevention (PTP) cohort, which follows individuals at risk for type 1 diabetes based upon family history and islet autoantibody status, also explored the relationship between BMI, BMI percentile, and insulin resistance (measured by HOMA-IR) with the progression from autoimmunity to diagnosis. Similarly, they found no association between any of these variables with the risk for developing type 1 diabetes (71). However, these studies lacked the ability to assess temporal trends. Recent, sophisticated analyses have examined the “cumulative excess BMI” (ceBMI) in the TrialNet PTP subjects, a calculated value describing the persistent elevation of BMI over time beyond the overweight threshold (BMI ≥ 25 kg/m^2^ in adults or ≥ 85^th^ percentile for age and sex in children). In children, ceBMI ≥ 0 kg/m^2^, indicating persistent overweight/obesity over time, was associated with a higher rate of progression from a single to multiple β-cell autoantibodies in children 9 years of age or older and without high-risk HLA genotypes (72). Furthermore, ceBMI ≥ 0 kg/m^2^ was also associated with a 63% greater risk to develop type 1 diabetes following islet autoimmunity in children, adjusted for age, sex, and autoantibody number (p=0.0009) (73). In adults, ceBMI also increased the risk for progression to type 1 diabetes, though only in certain age and gender cohorts, namely men over 35 years of age and women younger than 35 years age, suggesting some influence from sex hormones (74).

In further support of the accelerator hypothesis, insulin resistance likely adversely affects β-cell function in youth with type 1 diabetes and may promote immune activation in at risk individuals. Among pooled European cohorts of youth with type 1 diabetes, higher BMI at diagnosis was associated with greater decline in fasting C-peptide levels among teens 10–18 years at 1 year follow-up even when adjusting for glycemia, suggesting that BMI-related insulin resistance is contributing to β -cell dysfunction in this population (75). Resulting immune activation was suggested by a study examining T-cell autoreactivities to neuronal diabetes-associated autoantigens, which are typically observed early in type 1 diabetes pathology before antibodies emerge (76). Youth with the highest quintile of BMI or elevated waist circumference at onset of insulin dependent diabetes had higher islet associated T-cell autoreactivities, suggesting that T-cell autoimmunity may be promoted by visceral adiposity-associated insulin resistance. Furthermore, all but one youth without autoantibodies had evidence of T-cell autoimmunity, suggesting some immune activation among youth who might otherwise be classified as having type 2 diabetes.

Obese youth with new onset type 1 diabetes also have altered pro-inflammatory profiles which may contribute to the acceleration of their presentation. Adipocytes secrete a variety of pro- or anti-inflammatory adipokines which correlate with insulin resistance (77). Obese children with new onset type 1 diabetes are more likely to have higher pro-inflammatory markers (leptin, visfatin, chemerin, TNF-α, CRP) and lower anti-inflammatory markers (adiponectin, omentin) compared to non-obese peers with type 1 diabetes (78). While two or more autoantibodies at diagnosis, suggesting a higher degree of autoimmunity, are associated with higher adiponectin and lower leptin levels, these relationships were negated when adjusting for BMI, suggesting that adiposity is the primary driver of abnormal adipokines (79). Together, these studies examining ceBMI, autoimmune profiles, and inflammatory markers provide reasonable evidence to hypothesize that excessive weight gain may contribute to progression of islet autoimmunity to type 1 diabetes, perhaps through insulin resistance, altered adipokines and cytokines, and β-cell stress.

### Proposed Underlying Pathophysiology

Though no confirmed pathway exists linking obesity and the rising incidence of type 1 diabetes, different pathophysiological mechanisms may explain how obesity contributes to insulitis and autoimmunity, blurring the boundary with type 2 diabetes. Obesity-induced insulin resistance, insulin demand and inflammation are possible underlying mechanisms. Expanding adipose tissue through adipocyte hypertrophy leads to hypoxia and stress of the cellular endoplasmic reticulum (ER) (80). Subsequent adipocyte necrosis both activates and recruits tissue-specific macrophages, which expand in number from only 5–10% of stromal cells to 40–50%. These macrophages secrete a variety of pro-inflammatory cytokines (TNF-α, IL-6, and IL-1 β) and chemokines (CCL1, CXCL1, and CXCL2), which further activate the immune system (81). Locally, these pro-inflammatory signals impair insulin action and promote insulin resistance, ultimately leading to reduced glucose disposal and a failure to suppress both lipolysis and hepatic glucose production. In turn, insulin resistance, hyperglycemia, elevated free fatty acids, and cytokines promote additional ER and mitochondrial stress, perpetuating this cycle.

This milieu creates low-grade, chronic inflammation which may have downstream effects for β -cells. Insulin resistance and resulting inflammation are hypothesized to increase the β-cell secretory demand, placing added stress on the β-cell and resulting in local cytokine release (IFN-γ), neo-antigen formation, and β-cell apoptosis, thus triggering an immune response and insulitis (82, 83). In response to inflammatory signals, β-cells under stress may secrete chemokines, facilitating leukocyte recruitment and contributing to islet inflammation and destruction (84). Cytokines, specifically IL-1β and IFN-γ, may also trigger β-cell apoptosis directly *via* upregulation of the NF-κβ transcription pathway in islets (81). Interestingly, the pattern of immune cell activation in islets may differ between type 1 and 2 diabetes; adaptive immune cells predominate in type 1 compared to innate immune cells in type 2 diabetes (83). Additional studies are needed to further clarify the immune dysregulation that may occur in type 2 diabetes to underscore any underlying similarities in pathophysiological mechanisms. In sum, insulitis is likely driven by secretion of chemokines from activated immune cells and β-cells themselves in response to pro-inflammatory stimuli (cytokines, fatty acids), furthering leukocyte inflammation and contributing to β-cell death and, ultimately, type 1 diabetes (84). At present, it is unclear if obesity may be one environmental mechanism that perpetuates insulitis caused by an underlying unknown trigger, or if obesity itself could be a primary driver of insulitis; however, the data described above currently suggests the former.

### Type 2 Diabetes Genetic Risk

Genetic risk factors typically associated with type 2 diabetes may modulate the risk of autoimmune progression and development of clinical type 1 diabetes. The genetic risk factor most strongly associated with type 2 diabetes is TCF7L2 single nucleotide polymorphisms (SNPs). TCF7L2 SNPs are strongly associated with various aspects of type 2 diabetes, including insulin resistance, impaired insulin secretion, altered insulin processing, diminished suppression of glucagon by glucose, and increased fasting hepatic glucose release (85). These SNPs have been identified in children and adults with a single autoantibody at diagnosis of type 1 diabetes (86, 87) and seem to be inversely correlated with high-risk HLA genotypes (88). Those with these SNPs may present with a distinct phenotype characterized by less apparent islet cell damage. Oral glucose tolerance test stimulated glucose area under the curve was lower (p-0.0127) and area under the curve C-peptide was higher (p=0.008) in subjects with TCF7L2 SNPs identified at diagnosis (86). Furthermore, subjects with TCF7L2 SNPs may have altered progression from single to multiple autoantibodies, affected by both the initial antibody and the presence of overweight or obesity (89).

### Summary of Evidence

Taken together, obesity may augment the risk for autoimmune progression and development of type 1 diabetes when considering longitudinal assessments of excess weight. Hormonal differences in sex steroids likely play a role in potentiating this risk, given some findings in teens undergoing physiological insulin resistance of puberty (75) and differences observed in adults based upon sex and age (74). Proposed mechanisms include obesity-induced insulin resistance and inflammation increasing β-cell secretory demand, stress, and apoptosis, with autoimmune activation. Genetic factors traditionally associated with type 2 diabetes may also augment this risk. Perhaps most telling, the Diabetes Prevention Trial Type 1 Risk Score incorporates body mass index into its modern prediction model, highlighting the important, albeit complex, role of obesity in type 1 diabetes (90).

## Added Risk for Micro- and Macrovascular Complications

### Insulin Resistance and Cardiometabolic Markers

Premature CVD is a major cause of morbidity and mortality in adults with type 1 diabetes (91). Traditional risk factors for cardiovascular disease in the general population, including hypertension, dyslipidemia, and smoking, do not fully account for the increased CVD risk among those with type 1 diabetes. Insulin resistance emerged as the proposed link to explain the heightened risk for both microvascular and macrovascular complications (92). Applying a calculated measure of insulin resistance, the estimated Glucose Disposal Rate (eGDR) (93), to subjects in the longitudinal Pittsburgh Epidemiology of Diabetes Complications (EDC) study, increasing insulin resistance was associated with a higher risk of nephropathy (94), peripheral vascular disease (women only) (95), and coronary artery disease (96). This relationship in adult patients was further elucidated using data from the DCCT, where eGDR at baseline was significantly inversely associated with higher risk for nephropathy, retinopathy, cardiovascular events, and macrovascular disease (97). More recently, the Coronary Artery Calcification in Type 1 Diabetes (CACTI) study confirmed these findings using hyperinsulinemic euglycemic clamp data comparing adult subjects with type 1 diabetes to age, BMI, and physical activity-matched controls without diabetes (98). Insulin resistance correlated with the presence and progression of coronary artery calcifications, a surrogate marker for coronary artery disease and strong predictor of adverse outcomes. The subjects with type 1 diabetes had impaired insulin-mediated free fatty acid suppression, suggesting that lipotoxicity over time may be a potential mechanistic explanation.

Youth with type 1 diabetes may also have adipose, hepatic, and peripheral insulin resistance regardless of obesity and other features of metabolic syndrome (99). Early CVD factors are present in youth with type 1 diabetes despite their shorter duration of diabetes, also believed to be related to their decreased insulin sensitivity. In hyperinsulinemic euglycemic clamps, youth with type 1 diabetes are more insulin resistant compared to matched healthy peers and have lower functional exercise capacity, a marker of cardiovascular function and predictor of mortality (100). These findings were observed in normal weight youth with no features of Metabolic Syndrome, suggesting that some degree of insulin resistance is present regardless of adiposity. Using a clamp-derived measure, insulin sensitivity is inversely correlated with more atherogenic cardiovascular risk factors, including BMI z-score, total cholesterol, low density lipoprotein-cholesterol, blood pressure, and C-reactive protein (101), as well as arterial stiffness, measured by pulse wave velocity, in a longitudinal assessment (102).

### Obesity Compounds and Increases Risk for Complications

Though insulin resistance may account for much of the baseline cardiovascular risk profile in type 1 diabetes, the increased prevalence of obesity in this population likely augments this risk. Obese adults with type 1 diabetes have higher risk for micro- and macro-vascular comorbidities independent of glucose control (103). In the adult population from DCCT, the quartile with the highest weight gain in the intensively treated group also had higher BMI, blood pressure, triglycerides, total cholesterol, LDL-cholesterol, and apolipoprotein B compared to all other quartiles, indicative of a more atherogenic profile (104). These findings persisted in the EDIC follow-up of the DCCT and were associated with greater intima media thickness (105). Though the impact of obesity on cardiovascular complications is thought to be mediated by Metabolic Syndrome (e.g. hypertension, dyslipidemia), the CACTI study identified obesity as a risk factor for both the presence and progression of coronary artery calcifications independent of pre-existing metabolic abnormalities (106). How this translates to long-term cardiovascular morbidity and mortality is less clear (107). The Pittsburgh EDC study demonstrated that waist circumference, a surrogate of visceral adiposity, increases the risk for long-term mortality in type 1 diabetes (108). In the DCCT/EDIC studies, the rate of adverse cardiac events was initially similar between those who did or did not have excessive weight gain in the intensively treated group, with some divergence after 14 years, when the event rate in excessive weight gainers approached that of conventionally treated patients (109). The lack of a difference in events within 14 years of follow-up was attributed to both better management of cardiovascular risk factors (e.g. anti-hypertensive and lipid lowering medications) as well as the improved nutrition counseling this group received irrespective of overweight/obesity.

The potential compounding effect of obesity on insulin resistance and complications may appear as early as adolescence. Indeed, overweight and obese youth with type 1 diabetes are more likely to have coexisting hypertension, abnormal lipids, and elevated alanine aminotransferase compared to healthy weight peers (110, 111). In the SEARCH registry, youth with obesity and persistently elevated hemoglobin A1c over time had the highest risk for adverse cardiovascular markers and microvascular complications compared to those with either elevated hemoglobin A1c and normal weight or obesity with fairly-well controlled A1c (112). In clamp studies, our group found that obese youth with type 1 diabetes were more insulin resistant compared to non-obese peers with type 1 diabetes. Furthermore, insulin sensitivity correlated with cardiovascular risk factors, including pulse wave velocity, even when adjusting for the degree of obesity, suggesting that insulin resistance, augmented by excess weight, is the underlying factor contributing to cardiovascular complications (113). Overall, children do not meet ADA and ISPAD-determined clinical targets for glycemic control (114, 115), placing them at risk for long-term micro and macrovascular disease. Children with coexisting features of type 1 and 2 diabetes, or double diabetes, may be at heightened risk. Given the substantial changes in insulin therapy over time as a result of the DCCT with new analogs and continuous subcutaneous insulin infusion being used in children, the long-term impact on weight gain, insulin resistance, and cardiovascular health merits further study of intervention strategies.

## Therapeutic Approaches

Despite the more pronounced long-term risks of coexisting obesity and type 1 diabetes, there are limited therapeutics or guidelines to manage this condition beyond what has been traditionally available. One particular challenge for providers is the lack of a clinically meaningful definition for double diabetes which can guide treatment (116). Though glycemia is the most important predictor of outcomes, the degree of insulin resistance associated with obesity and type 1 diabetes is also important in predicting future complications, and this is not addressed by insulin titration alone. Escalating insulin doses to achieve glycemic goals may also further compound weight gain, exacerbating obesity-related complications. Adequate counseling on a healthy diet and adequate physical activity should be paramount in the care of these patients, following recommended guidelines from leading diabetes organizations (43, 46). In addition, the use of targeted nutritional therapies or pharmaceuticals for the treatment of type 2 diabetes have been proposed as adjuvant treatments for children and adults with obesity complicating type 1 diabetes; the evidence for these therapies is summarized in **Table 1**.

**Table 1:** Summary of Evidence for Adjunctive Pharmaceuticals in Type 1 Diabetes.

Drug Category	Mechanism	Summary of Evidence
Biguanides (Metformin)	Improves insulin sensitivity by blocking hepatic gluco-neogenesis	Adequately powered studies in youth have found no improvement in hemoglobin A1c (117–119) May result in a modest reduction in daily insulin dose and BMI (117, 119–121) Possible cardioprotective effects, though evidence is limited (119, 122, 123)
GLP-1 agonists	Stimulates insulin release and inhibits glucagon secretion in a glucose-dependent manner; induces satiety	Across multiple trials in adults, small improvement in hemoglobin A1c (-0.21%) (124) Mean weight loss of approximately 3.5 kg (124) May lower daily bolus insulin (124) No available studies in youth with type 1 diabetes
DPP4-inhibitors	Blocks degradation of endogenous GLP-1	Across multiple trials in adults, no improvement in hemoglobin A1c, BMI, or insulin dose (125, 126) No available studies in youth with type 1 diabetes
SGLT 1/2 inhibitors	Blocks sodium-glucose transporter in the proximal tubule of the kidney resulting in glycosuria	Across multiple trials in adults, small reduction in hemoglobin A1c (-3.9%) (127) Daily insulin dose reduced by ~10% (127) Body weight reduced by ~4% (127) No available studies in youth with type 1 diabetes examining change in weight (128)

### Targeted Nutritional Therapies

Diet is an essential component to type 1 and 2 diabetes management and is necessary to achieve optimal glycemic control (129). Both the ADA and ISPAD promote nutrition counseling and education that is tailored to the unique psychosocial and cultural needs of that family (43, 130). As a guide, children should receive approximately 45–50% of their energy from carbohydrates, 15–20% from protein, and <35% from fat (with saturated fat <10%) (130). The dietary recommendations must be commensurate with the insulin regimen, as certain approaches (e.g. sliding scales/algorithms) require fixed- macronutrient intake for each meal time. The diet for children and adolescents with type 1 diabetes is not only important for glycemic control, but also for appropriate growth and development while minimizing unnecessary weight gain. In adults with type 1 diabetes, both the Mediterranean and Dietary Approaches to Stop Hypertension (DASH) diets have been shown to promote weight loss as well as optimize glucose levels (131).

The low carbohydrate or very low carbohydrate (ketogenic) diets have gained recent popularity as part of the treatment for type 1 diabetes. They require significant carbohydrate restriction, at times to no more than 20–70 g/day. The studies examining the effectiveness of this diet are heterogeneous and often small, limiting their interpretation. Some evidence suggests there may be a beneficial reduction in hemoglobin A1c, daily insulin dose, and body weight mostly in adults, though this may be at the expense of insufficient insulin, dyslipidemia or increased hypoglycemic events (132). Use of this diet in children, especially if normal weight, is highly controversial, as carbohydrate intake and appropriate insulin dosing are critical for growth. A case series documented poor growth, significant fatigue, and adverse lipid profiles in six children with type 1 diabetes on a low carbohydrate diet (133). At present, the low carbohydrate diets are not recommended for youth with type 1 diabetes, including as treatment for obesity. The relationship between any of these diets and insulin resistance has not been studied. Similarly, studies have not examined whether weight control in children at risk for type 1 diabetes can help prevent the onset of the disease.

### Metformin

Metformin is considered first line therapy in both adolescents and adults with type 2 diabetes. Its mechanism is believed to be improved insulin sensitivity by inhibiting hepatic gluconeogenesis, resulting in reduced hepatic glucose output, and increasing peripheral uptake of glucose in the muscle (134). Metformin has been proposed as an adjunct therapy in both youth and adults with type 1 diabetes to address peripheral insulin resistance regardless of weight; relevant pediatric studies are summarized in **Table 2**. Numerous randomized controlled trials have attempted to explore the efficacy of metformin in this context. Initial studies examined adolescents with poor glycemic control, only some of whom were overweight and obese, with few finding a modest improvement in hemoglobin A1c (120, 121, 135, 136) and others finding no difference (137). These studies were small, of variable duration, and frequently used non-standard doses of metformin, limiting their interpretation. An adequately powered trial using low dose metformin (1,000 mg daily) in adolescents with sub-optimal glycemic control (hemoglobin A1c >8.5%), only approximately one-third of whom were overweight or obese, found no difference in hemoglobin A1c levels from baseline to six months (117). Two additional studies specifically examined use of metformin in overweight or obese adolescents (118, 119). A large randomized trial found that though 2,000 mg of daily metformin led to a small decline in hemoglobin A1c compared to placebo at the mid-way point, at 6 months there was no difference in hemoglobin A1c compared to placebo (119).

**Table 2:** Summary of Clinical Trials of the Use of Metformin in Youth with Type 1 Diabetes.

Citation	N	Intervention	Comparison?	Duration	Effect on HbA1c	Effect on dailyInsulin Dose	Effect on Weight
Gomez et al. J Pediatr Endocrinol Metab. 2002. (135)	10	Variable dose metformin	No	6 months	Decrease by 11% of baseline	No change	No change
Hamilton et al. Diabetes Care. 2003. (120)	27	Weight-based dose metformin (up to 2000 mg/day)	Placebo	3 months	-0.6% (p=0.03)	-0.16 units/kg (p=0.01)	No change
Urakami et al. Pediat Int. 2005. (121)	9	500-750 mg twice daily metformin	No	12 months	-1.1% (p<0.01)	-6.7 units (p<0.01)	-0.7 kg/m^2^ (p<0.05)
Nadeau et al. Pediatr Diabetes. 2015. (117)	74	1000 mg daily metformin	Placebo	6 months	No change	No change	No change
Nwosu et al. PLoS One. 2015. (118)	28	1000 mg daily metformin	Placebo	9 months	No change	No change	No change
Libman et al. JAMA. 2015. (119)	140	2000 mg daily metformin	Placebo	6 months	No change	-0.1 units/kg (p<0.001)	-0.1 BMI z-score (p<0.001)

Despite the lack of clinical change in hemoglobin A1c over the course of these trials, many studies demonstrated a reduction in body mass index (117, 119, 121), waist circumference (117), and insulin dose per unit of body weight (117, 119–121). Indeed, the aforementioned large randomized trial of 2,000 mg metformin daily in obese adolescents demonstrated that approximately one quarter of the intervention group had at least a 25% reduction in their total daily insulin dose per unit body weight (compared to 1% in placebo group p=0.003) and at least a 10% reduction in their BMI z-score (compared to 7% in placebo group p=0.01) (119). In addition, there was a small reduction in total body fat by approximately 2 kg as determined by dual-energy x-ray absorptiometry in the metformin group compared to placebo (p<0.001).

Taken together, these findings have mirrored those in adults, which similarly have found no improvement in hemoglobin A1c, though reductions in insulin dose and body weight may occur (122, 138). The noted reductions in insulin dose were thought to imply improved insulin resistance. Two studies examined this directly using hyperinsulinemic euglycemic clamps, finding improvement in both whole-body insulin sensitivity and peripheral insulin resistance in those adolescents taking metformin (136, 139). How this actually relates to long-term cardiovascular complications is less clear, as studies are inconclusive on whether the addition of metformin impacts intermediate measures of hypertension, lipids, inflammatory markers, or carotid intima media thickness (119, 122). Through the use of advanced magnetic resonance imaging techniques, metformin may lead to some benefit in measures of aortic pulse wave velocity and wall sheer stress among youth, suggesting cardioprotective effects (123). The potential downstream benefits, particularly as pertains to the risk for micro- and macro-vascular complications, warrant further investigation.

At present, use of metformin is not recommended by the ADA for youth with type 1 diabetes (43), and though it is discussed as a consideration in the guidelines for adults with type 1 diabetes, it is not explicitly recommended (140). Real-world studies evaluating how often metformin is used within a type 1 diabetes population and which patients receive this adjunct therapy are limited. Among both the T1D Exchange and DPV registries, including close to 50,000 patients (both youth and adults), metformin was used in about 3.5% of individuals in the T1D Exchange and only 1.3% in the DPV (141). Adjuvant therapy was rare in youth under 13 years of age. An additional analysis of the DPV registry examined 525 youth with type 1 diabetes treated with metformin in addition to insulin compared to over 57,000 youth on insulin alone (142). In this observational study, the children treated with metformin tended to be slightly older and were more commonly female. Median BMI z-score of the metformin-treated youth was +1.86 (+1.33 to +2.58) compared to a median BMI z-score of +0.51 (−0.12 to +1.15) in youth on insulin alone (p<0.001). Furthermore, hemoglobin A1c was significantly higher among those on metformin, as was the presence of comorbidities, including polycystic ovary syndrome, hypertension, elevated liver enzymes, and dyslipidemia. Those youth treated with metformin required a significantly higher insulin dose per unit body weight than those on insulin alone. Over time, the metformin-treated group had modest improvements in BMI and insulin dose. The study concluded that metformin is used rarely in youth with type 1 diabetes outside of the research setting, most often in obese female adolescents, perhaps to treat concomitant polycystic ovary syndrome, and the benefits of this therapy are not fully realized.

### GLP-1 Receptor Agonists and DPP-4 Inhibitors

Glucagon-like-peptide-1 (GLP-1) is an incretin hormone secreted by the gut which stimulates insulin release and inhibits glucagon secretion in a glucose-dependent manner, resulting in control of meal-induced glycemic excursions (143). GLP-1 also delays gastric emptying and acts centrally to decrease appetite (144), which can help to promote weight loss. GLP-1 receptor signaling may also support islet health by inhibiting β-cell apoptosis, promoting proliferation of β-cells, and reducing ER stress, with the net result of preserving or enhancing residual function; however, restoration of β-cell function has not been proven in human studies of islet transplantation or type 1 diabetes (145). GLP-1 is rapidly degraded by the dipeptidyl peptidase-4 enzyme (DDP-4), limiting the longevity of its effect. Both GLP-1 receptor agonists and DPP-4 inhibitors are recent advances for the treatment of type 2 diabetes, and in the case of GLP-1 agonists, for obesity in the absence of diabetes. In addition to myriad studies in adults, the GLP-1 agonist, Liraglutide, has been shown to be effective in reducing hemoglobin A1c in youth with type 2 diabetes (146) and promoting weight loss in obese adolescents with no evidence of dysglycemia (147).

While this treatment has not yet been studied in obese children with type 1 diabetes, there is some evidence supporting the use of GLP-1 agonists in adults with type 1 diabetes. A meta-analysis examined the net impact of GLP-1 agonists on glycemic control, weight, and insulin dose in seven randomized controlled trials in 206 adult subjects with type 1 diabetes also treated with insulin over study durations of 3–15 months (124). Overall, there was a net, albeit modest, improvement in hemoglobin A1c by −0.21% (95% CI −0.40, −0.02, p=0.03), without increased risk of hypoglycemia, and mean weight loss of 3.53 kg (95% CI −4.86, −2.19, p<0.05) in adults taking GLP-1 agonists compared to placebo. In the few studies that examined total daily insulin dose, specifically differentiating bolus and basal insulin, use of a GLP-1 agonist was associated with a 6% reduction in daily bolus insulin dose (95%CI, −10%, −2%, p=0.001) with no change in absolute daily insulin dose. Taken together, the authors suggest a potential benefit to adding GLP-1 to insulin therapy in adults with type 1 diabetes and offer different mechanisms by which GLP-1 may mediate these improvements. Two explanations may be improved insulin sensitivity (148) and/or reduced appetite and carbohydrate intake (149), resulting in decline in bolus insulin requirements and weight loss (124). In contrast, DPP-4 inhibitors are less promising. Two meta-analyses examined the efficacy and safety of DPP-4 inhibitors in adults with type 1 diabetes; neither found any significant improvement in hemoglobin A1c, BMI, or insulin dosage with treatment, though significant heterogeneity among studies limited the analyses (125, 126).

Altogether, adult studies have identified a slight improvement in glycemic control, weight, and bolus insulin dose among patients with type 1 diabetes on combined therapy of insulin and GLP-1 agonists, but not DPP-4 inhibitors. The therapeutic mechanisms underpinning these improvements are poorly understood based upon the available studies, though the theoretical advantage to these medications may predominantly be weight loss in the setting of overweight/obesity, which may improve insulin sensitivity. To date, no studies have examined the use of either of these therapies in youth with type 1 diabetes, and they are not currently recommended for this population.

### SGLT-1 and 2 Inhibitors

Sodium-glucose cotransporter (SGLT) inhibitors are another fairly recent therapeutic advancement approved for the treatment of type 2 diabetes which have been proposed as an adjunct treatment for type 1 diabetes. These medications block SGLT-2 in the proximal tubule of the kidney, resulting in glycosuria and natriuresis; some medications also block SGLT-1, which can delay glucose absorption from the intestinal tract (150). In adults, SGLT inhibitors are believed to have added cardioprotective effects (151). A meta-analysis examined 10 randomized controlled trials of both SGLT-2 and combined SGLT-1/2 inhibitors, including over 5961 patients with type 1 diabetes with follow-up of 12–52 weeks (127). Across pooled studies, these medications resulted in a reduction in body weight of approximately 4% and associated reductions in hemoglobin A1c by −0.39% (95% CI −0.43 to −0.36) and fasting glucose by −1.13 mmol/L (95% CI −1.36 to −0.90). In addition, total daily insulin dose, basal insulin dose, and short acting insulin dose were all reduced by approximately 10%. Thus far in youth with type 1 diabetes, one study has examined safety and effect of SGLT-2 inhibitors, though did not comment on any effect on weight (128).

These medications essentially compensate for some food indiscretions by lowering the renal threshold, promoting the excretion of excess glucose following a meal. By reducing hyperglycemia, these drugs likely facilitate weight loss; both are proposed mechanisms for improved insulin sensitivity in adults with type 2 diabetes (152, 153). No investigations have evaluated whether the same metabolic effects are present in individuals with type 1 diabetes regardless of adiposity, and use of these medications in this population remains fairly cautionary due to the increased risk for euglycemic ketoacidosis (127, 154). Similar to the therapies described before, SLGT inhibitors are not currently recommended for adjuvant therapy in pediatric type 1 diabetes with obesity.

In all, the options to treat obese youth with type 1 diabetes remain limited. The prevention and management of obesity continues to rely upon early, consistent, and individualized dietary counseling both at diagnosis and at least annually at follow-up appointments. No therapeutic options are currently approved for this purpose, and future research is needed to understand which therapies might be safe and effective in weight reduction, optimizing glycemic control, and in preventing long-term complications.

## Discussion and Future Directions

Overweight and obesity are increasingly common in youth and adults with type 1 diabetes, perhaps a consequence of changing cultural practices surrounding diet, nutrition and exercise over the past several decades. Genetic risk and environmental factors may lead to coexisting features of type 1 and 2 diabetes, with added implications for both pathogenesis and long-term health. Data supporting these outcomes led to the concept of double diabetes. In the setting of widespread use of intensive insulin therapy, poor dietary choices and over-treatment of hypoglycemia may both contribute to weight gain. Further study is needed to understand whether use of new technologies, particularly closed loop hybrid insulin pumps and automated insulin delivery systems, may help reduce the risk of hypoglycemia and thus result in decreased weight gain by lessening the need for treatments with added food intake.

It is possible that obesity has a role in potentiating the risk for type 1 diabetes, similarly to type 2 diabetes. Obesity causes augmentation of insulin resistance, which may increase both insulin demand, stressing the islets. Though longitudinal studies have examined the role of excessive weight gain in autoimmune development and progression, future research is needed to better elucidate the mechanisms by which obesity contributes to increased stress on the insulin secretory functions and inflammation, possibly leading to or exacerbating β-cell pathology.

There is limited evidence for any beneficial effect of currently available adjuvant therapies apart from lifestyle adjustments, and these all need further investigation for both the delay of type 1 diabetes and the prevention of complications in those with double diabetes. Better measurement systems are needed to identify those with significant insulin resistance who may be at higher risk for complications that are easily adaptable for busy clinical settings. Additional investigations are needed to examine whether the use of targeted therapies for weight loss can also modulate inflammation and insulin resistance and reduce the long-term risk for cardiovascular complications. Given our current fund of knowledge, it is critical to consider how we counsel patients and families about excess weight gain for children diagnosed with type 1 diabetes to prevent double diabetes in adolescence and adulthood. Perhaps most important are practical, evidenced-based strategies to promote lay awareness of the dangers of weight gain in the type 1 diabetes population and focus efforts on obesity prevention.

## Author Contributions

All authors contributed to the conceptualization of this manuscript and identified source articles. CM drafted the manuscript. All authors contributed to the article and approved the submitted version.

## Funding

The work was funded by Mentored Patient-Oriented Research, National Heart, Lung and Blood Institute, NIH 5K23HL085287-03 (Primary Investigator: IL), and UPMC Children’s Scholar Award (Primary Investigator: CM).

## Conflict of Interest

IL serves as Pediatric Type 2 Diabetes Expert Panel member for Novo Nordisk.

The remaining authors declare that the research was conducted in the absence of any commercial or financial relationships that could be construed as a potential conflict of interest.

## Abbreviations

BMI, body mass index; DPV, Diabetes Patienten Verlaufsdokumentation registry; ADA, American Diabetes Association; ISPAD, International Society for Pediatric and Adolescent Diabetes; DCCT, Diabetes Control and Complications Trial; EDIC, Epidemiology of Diabetes Interventions and Complications study; PTP, Pathway to Prevention; ceBMI, cumulative excess BMI; HOMA-IR, homeostatic model assessment of insulin resistance; ER, endoplasmic reticulum; SNPs, single nucleotide polymorphisms; CVD, cardiovascular disease; eGDR, estimated glucose disposal rate; EDC, Epidemiology of Diabetes Complications study; CACTI, Coronary Artery Calcification in Type 1 Diabetes; GLP 1, glucagon-like peptide 1; DPP4, dipeptidyl peptidase 4; SGLT, sodium glucose cotransporter.
